# Modification of External Fixator in Cross-Leg Flap: A Stabilizing Solution for an Optimal Outcome

**DOI:** 10.7759/cureus.112621

**Published:** 2026-07-13

**Authors:** Rahul Dubepuria, Deepak Krishna, Chandrika Chityala, Varsha R Choudhary

**Affiliations:** 1 Department of Burns and Plastic Surgery, All India Institute of Medical Sciences, Bhopal, Bhopal, IND; 2 Department of Obstetrics and Gynaecology, Gandhi Medical College, Bhopal, IND

**Keywords:** cross-leg flap, external fixator, lower limb reconstruction, monophasic flow, pedicled flap reconstruction, soft tissue defect

## Abstract

Background: Lower limb defects with single-vessel supply and with monophasic flow are difficult to reconstruct due to paucity of coverage options. The cross-leg flap remains a reliable salvage option in these settings. But maintaining the position for three weeks is cumbersome for both the patient and the flap. Any movement of the lower limb generates a shearing force at the flap inset, predisposing to partial flap necrosis and dehiscence. An external fixator helps both the stable fixation of the lower limbs and the prevention of stretch in the flap. We aim to describe a modification in the conventional external fixator to prevent maceration of the lower limbs and the flap, and also facilitate ease of dressing.

Materials and methods: We assessed eight patients retrospectively who underwent cross-leg flap for post-traumatic leg defects, where an external fixator was applied for better flap stabilisation.

Results: All patients were male with post-traumatic soft tissue defects in various regions of the leg and foot, with variable underlying vital structures exposed. The average age of the patients was 41 years, with a range of 16-55 years. An external fixator was applied in all cases, with a modification of an additional tubular rod used at the base to prevent the legs from contacting the bed, thus preventing maceration and difficulty in dressing. All flaps survived well, but with partial necrosis of the distal margin in two patients, which was managed with debridement and advancement of the flap. The donor site was skin grafted.

Conclusion: External fixator is a reliable option for stabilising lower limbs in cases of cross-leg flap reconstruction. With the modification, it not only stabilises the lower limbs but also helps in preventing maceration and increasing airflow.

## Introduction

Extensive injuries to the lower leg are common in high-velocity injuries and pose a challenge to plastic and orthopaedic surgeons. Severe trauma with composite fractures needs bony stabilisation as well as soft tissue coverage. A cross-leg flap is the last resort for reconstruction when local and free flaps are difficult to use due to concomitant vascular injury or the unavailability of local soft tissue to cover the defect [1]. However, it is possible to transplant a pedicle from the medial part of the calf to any area of the foot and leg without manipulating extremities into a difficult position [2]. At the same time, maintaining position after a cross-leg flap is cumbersome and associated with several problems, including pressure injuries, wound dehiscence, and dressing difficulties [3]. To overcome this, the trend now is the application of an external fixator rather than a plaster cast to stabilise both legs.

By definition, an external fixator is a medical device that provides stability and support to the affected limb by using pins or wires inserted into the bone and connected to an external frame. This device allows for precise control over the position and movement of the limb. An external fixator contributes to cross-leg flap success by: (i) Minimising movement at the flap inset, thereby promoting flap survival, (ii) Eliminating the need for cumbersome casts, improving patient comfort, and (iii) Enabling easier wound access for regular dressing changes [4]. The application of a proper external fixator system is necessary to stabilise the legs and manage the associated problems, thereby achieving optimal outcomes.

In our study, after counselling the patients and giving them the necessary education and psychological support, we used bilateral and biplanar fixators, in which an extra set of rods was attached to a conventional fixator to offload both limbs, thereby preventing pressure injuries [5] and pain over the heel and cutting the need for leg lifting during dressing.

## Materials and methods

This was a retrospective analysis of patients who sustained road traffic accident-related soft tissue defects and burn injury involving various regions of the leg and foot, with exposure of underlying vital structures and either monophasic vascular flow or a single-vessel limb in the Department of Burns and Plastic Surgery at All India Institute of Medical Sciences, Bhopal, Madhya Pradesh, India, from January 2024 to December 2025. The study was approved by the Institutional Human Ethics Committee-Student Research, All India Institute of Medical Sciences, Bhopal (registration number: Ihecsr/aiimsbpl/July23/127). Informed consent was obtained before administering the treatment.

Study population

Inclusion criteria included patients who underwent cross-leg flap surgery that was stabilized using the modified external fixator technique, who were willing to comply with postoperative immobilization, follow-up protocol, and flap division, and who provided written informed consent (or guardian consent for minors, if included).

Exclusion criteria included patients who underwent conventional immobilization methods instead of the modified external fixator and in whom the modified external fixator could not be applied because of anatomical or orthopedic contraindications, patients who had incomplete medical records or inadequate documentation, and patients who did not give consent for the surgery. 

A total of eight patients were included, of whom seven had RTA, and one had burn injuries, as mentioned above.

Data collection

Demographic characteristics, defect location, surgical planning details, and vascular status using colour Doppler were recorded and analysed. Colour Doppler examination was performed preoperatively. Monophasic arterial flow was defined as a low-resistance, single-phase waveform with loss of the normal triphasic or biphasic pattern, indicating significant distal arterial insufficiency and influencing the reconstructive strategy. 

Surgical procedure

Perioperative Protocol

Flap monitoring: Clinical assessment of flap colour, temperature, capillary refill, and bleeding on pinprick were done at regular intervals.

Dressing schedule: First dressing was applied on Postoperative Day (POD) 3-5, then on every alternate day.

Flap division: This was performed after three weeks, once flap integration was clinically satisfactory.

Technique of External Fixator application

For fixation, four sets of pins were used. After recreation of the defect following debridement of the wound, the flap was marked based on the planning in a reverse manner.

Set 1: Three 4.5-mm Schanz pins were inserted into the injured limb through the medial aspect of the tibia (tibial shin). Of these, two pins were placed proximal to the fracture site, while one was inserted distal to the fracture. In the contralateral (uninjured) limb, two 4.5-mm Schanz pins were used, with one placed in the proximal tibia and the other in the distal tibia. Pins were placed obliquely to avoid colliding with the pins on the other limb. After this, the flap was elevated, and the two limbs were brought together for insetting.

Set 2: One tubular connecting rod was placed in each limb, which stabilised the vertical pins with universal AO clamps. Once the horizontal connecting rods were connected in the desired position, the inset of the flap was completed.

Set 3: Two horizontal connecting rods were placed to maintain both legs in the relevant position, and thus, the two legs were brought together.

Set 4: Two oblique tubular rods were attached to the distal set of Schanz pins and connected to two vertical tubular rods, one on each side. These vertical rods elevated the entire external fixator construct. To enhance the stability of the construct on the bed, the two vertical rods were interconnected with a long horizontal connecting rod, creating a stable and level base for the external fixator framework. It prevented the splaying of kickstand rods and provided additional stability. A wooden plank was placed beneath the framework to maintain elevation and prevent the cushioning effect of the mattress. The inset of the flap was done after fixation of the external fixator on both legs.

## Results

A total of eight patients with complex lower limb soft tissue defects underwent cross-leg flap reconstruction during the study period and fulfilled the eligibility criteria. Complete demographic and operative details are summarised in Table 1. All patients were male, aged 16-55 years (mean age: 41 years). The most common aetiology was RTA, accounting for seven cases (87.5%), and for the remaining patient, it was thermal burn (12.5%). Injury site was at the mid to lower third of the leg (n=3, 37.5%), mid third of the leg (n=3, 37.5%), and two cases had heel defects (25%). Exposed or segmental loss of the tibia was seen in six cases (75%), and exposed calcaneum was seen in two cases (25%), with associated fibular fracture/fragments in two cases (25%). Transverse cross-leg flap based on posterior tibial artery (PTA) perforators was used in six cases (75%), and inferiorly based cross-leg flap using PTA perforators was used in two cases (25%). Preoperative colour Doppler assessment demonstrated compromised vascularity in all patients, with monophasic flow in the anterior tibial artery (ATA) in all eight cases (100%), monophasic flow in the PTA in five cases (62.5%), and monophasic flow in the dorsalis pedis artery (DPA) in four cases (50%). We observed that cross-leg flap reconstruction proved useful in patients with poor vascularity and exposed critical structures such as the tibia and calcaneus. The PTA perforator-based transverse cross-leg flap was the most commonly employed flap in our study.

**Table 1:** Patient demographics, defect description, etiology, surgical plan, underlying exposed structure, time of division, vascularity status and complications RTA: road traffic accident; PTA: posterior tibial artery; ATA: anterior tibial artery; DPA: dorsalis pedis artery; M: male

Case no	Age	Sex	Etiology	Site and size of Defect	Underlying exposed structures	Type of flap	Vascularity status	Time of division of cross-leg flap	Complication
1	55 years	M	RTA	Mid to lower third of right leg; 18 x 9 cms	Segmental defect of tibia	Transverse cross-leg flap (PTA perforator)	Monophasic flow in ATA, PTA and DPA	21 days	Nil
2	52 years	M	RTA	Mid third of right leg; 21 x 8 cms	Segmental defect of tibia	Transverse cross-leg flap (PTA perforator)	Monophasic flow in ATA and DPA	21 days	Nil
3	16 years	M	RTA	Left heel (previously operated with loss of regional flap); 12 x 7 cms	Exposed calcaneum	Transverse cross-leg flap (PTA perforator)	Monophasic flow in ATA and PTA	21 days	Nil
4	53 years	M	Thermal Burn	Mid to lower third of left leg; 18 x 6 cms	Exposed tibia	Transverse cross-leg flap (PTA perforator)	Monophasic flow in ATA	21 days	Partial distal flap necrosis
5	40 years	M	RTA	Lower third of right leg; 12 x 5 cms	Exposed tibia	Inferiorly based cross-leg flap (PTA perforators)	Monophasic flow in ATA and PTA	21 days	Nil
6	36 years	M	RTA	Mid third of left leg; 14 x 7 cms	Segmental loss of tibia	Transverse cross-leg flap	Monophasic flow in ATA and DPA	21 days	Partial distal flap necrosis
7	46 years	M	RTA	Left heel; 10 x 6 cms	Exposed calcaneum, closed fracture of tibia and fibula	Inferior-based cross-leg flap	Monophasic flow in ATA and PTA	21 days	Nil
8	28 years	M	RTA	Mid to lower third of right leg; 15 x 6 cms	Exposed tibial fragments, fractured fibula	Transverse cross-leg flap	Monophasic flow in ATA and DPA	21 days	Nil

Case 1

A 55-year-old male patient presented following an RTA with injuries to the left leg, resulting in a soft tissue defect measuring 18 × 9 cm over the anteromedial aspect of the mid and lower thirds of the leg, associated with an approximately 5-cm segmental loss of the tibia. The patient presented three months after the initial injury, during which period the wound had been managed conservatively with regular dressings at an outside facility, leading to healthy granulation tissue formation over the wound bed.

**Figure 1:**
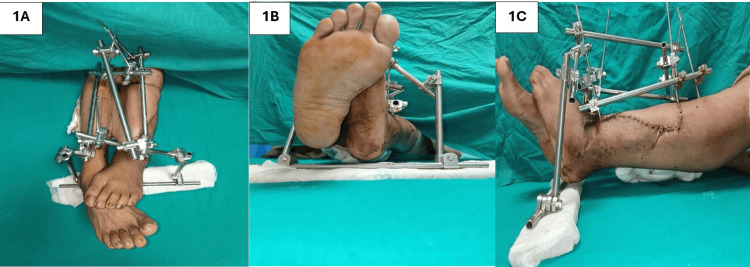
Case 1 (A) Cross-leg flap with external fixator superior view, (B) base view showing the elevation of fixator with wooden plank underneath it to prevent cushioning effect, and (C) Left lateral view showing one set of tubular rods used to elevate the frame.

In view of the anticipated need for future bony reconstruction, soft tissue coverage with a cross-leg flap was planned. A medially based transverse cross-leg flap was designed on the contralateral leg using reverse planning. The flap was elevated in the subfascial plane, extending up to 2 cm posterior to the medial border of the tibia while maintaining a width-to-length ratio not exceeding 1:2. Following application of the external fixator, the flap was inset into the defect, and the donor site was covered with a split-thickness skin graft (Figure 1).

Case 2

A 52-year-old male patient sustained trauma to the right leg, resulting in fractures of the tibia and fibula associated with extensive soft tissue loss over the mid and lower thirds of the anterior aspect of the leg. Initial management included skeletal stabilisation with plate-and-screw fixation and application of an external fixator in the same operative setting. The patient presented to our institution two months later with exposed hardware and exposed tibia involving the mid and lower thirds of the right leg. The exposed tibial segment appeared nonviable, with yellow discolouration and multiple fenestrations, and the implant was visibly exposed.

**Figure 2:**
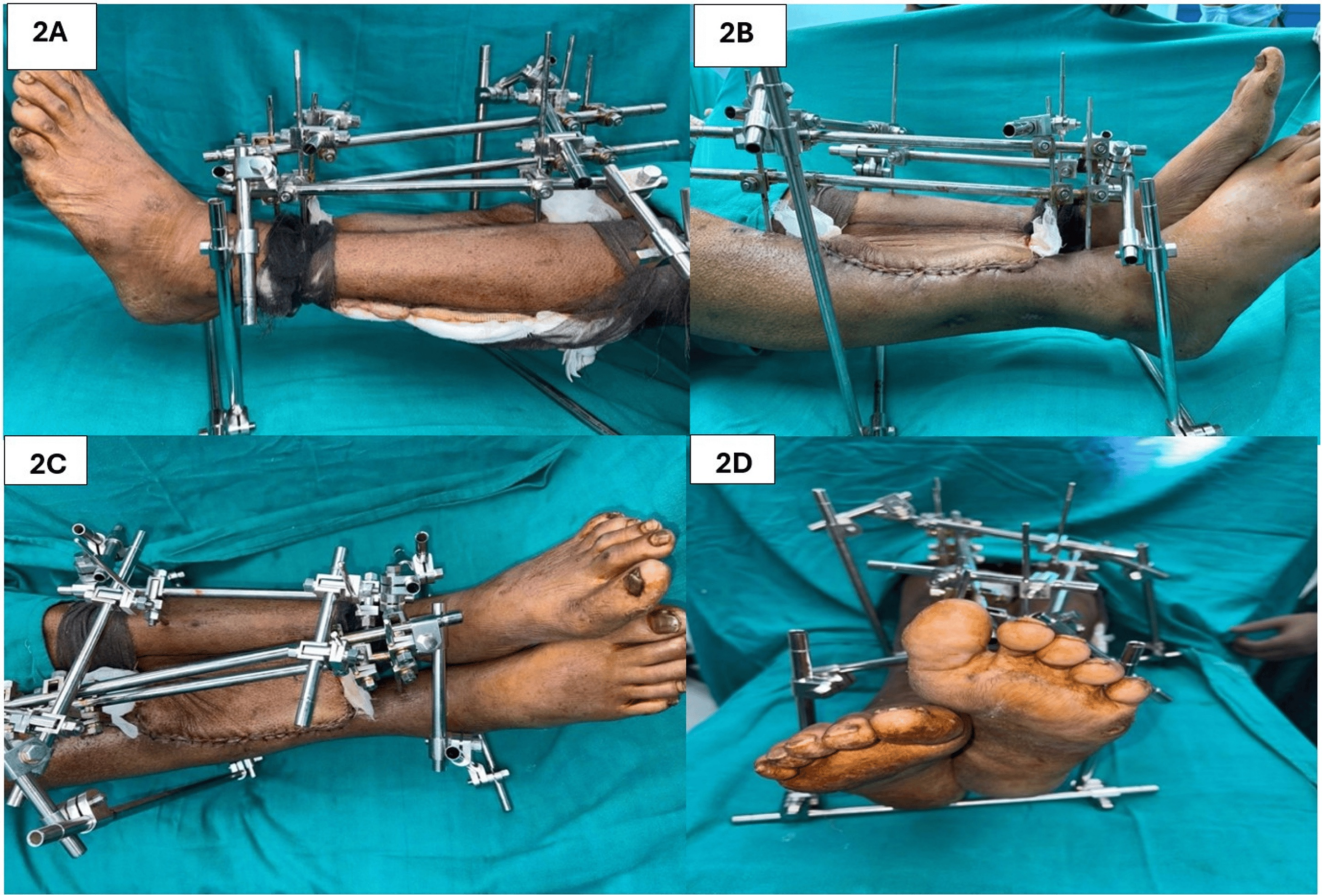
Case 2 (A) Cross-leg flap with external fixator left lateral view, (B) right lateral view showing two set of tubular rods used to elevate the frame, and (C) superior view and (D) base view showing the elevation of fixator

Following the assessment, the orthopaedic team removed the exposed implant along with a 12-cm segment of tibia. Subsequent thorough wound debridement resulted in a soft tissue defect measuring 21 × 8 cm. Reconstruction was performed using a PTA perforator-based transverse cross-leg flap measuring 22 × 12 cm with a 4-cm skin bridge, harvested from the contralateral leg. Bilateral lower limbs were stabilized using an external fixator with both knees maintained in slight flexion (Figure 2).

Case 3

A 16-year-old male patient, with a history of RTA four years earlier, presented with recurrent ulceration, unstable scarring, and flexion deformity of the left foot. Following the initial injury to the left heel, the patient underwent local flap reconstruction at a private hospital; however, the flap subsequently became necrotic, and the wound eventually healed by secondary intention.

**Figure 3:**
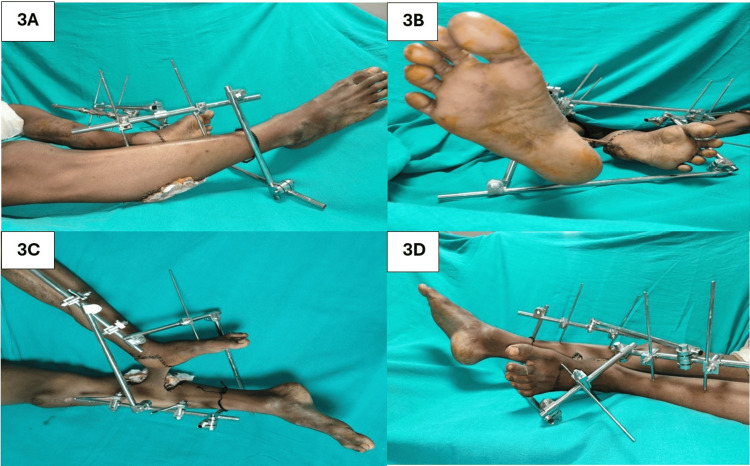
Case 3. (A) Cross-leg flap with external fixator left lateral view showing one set of tubular rods used to elevate the frame and (B) base view, (C) superior view, and (D) right lateral view showing the elevation of fixator.

Owing to persistent symptoms and instability of the scar, reconstruction using a PTA perforator-based transverse cross-leg flap was planned. After excision of the unstable scar and associated calcaneal spur, the resultant defect measured approximately 12 × 7 cm. A transverse cross-leg flap measuring 15 × 7 cm was harvested based on PTA perforators from the contralateral limb. Postoperatively, an external fixator was applied to maintain limb positioning, with the left knee kept in flexion and the left hip in external rotation (Figure 3).

Case 4

A 53-year-old male patient presented with a history of thermal burn injury and a soft tissue defect involving the anterior aspect of the middle and distal thirds of the left leg, associated with exposure of the tibia.

**Figure 4:**
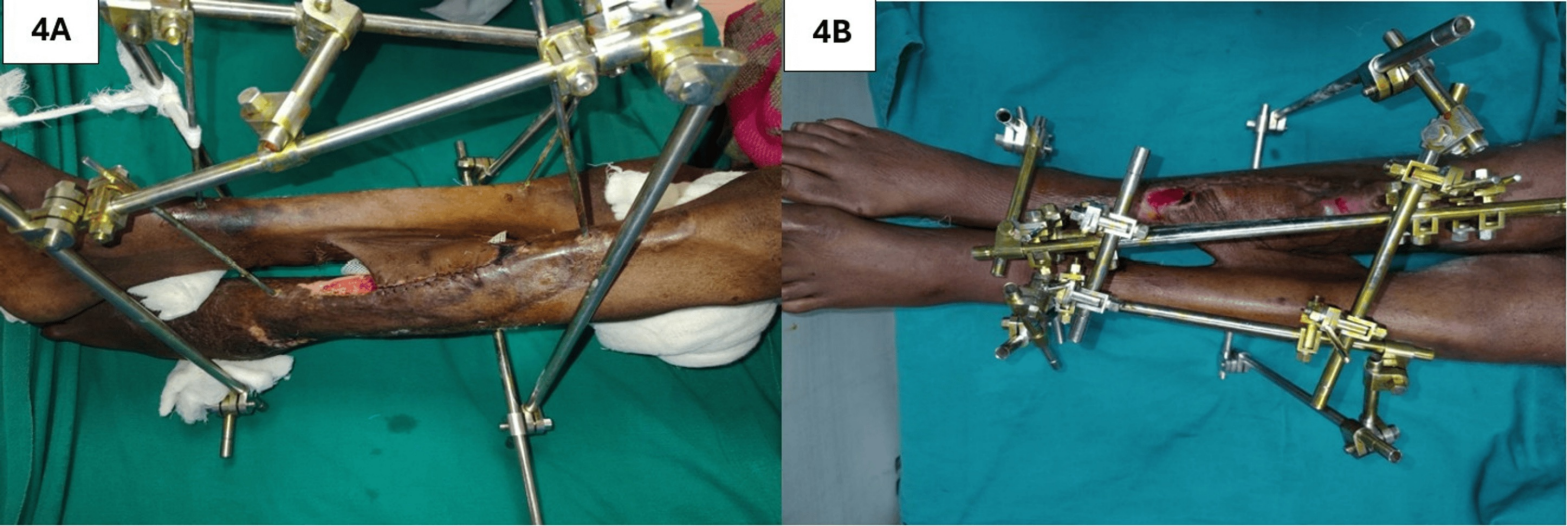
Case 4. (A) Cross-leg flap with external fixator left lateral view showing two set of tubular rods used to elevate the frame, (B) Superior view

Reconstruction of a defect of size 18 x 6 cms was performed using a PTA perforator-based transverse cross-leg flap harvested from the contralateral leg. Postoperatively, limb positioning and flap stability were maintained with the application of an external fixator. There was distal flap necrosis of 3 cms which was managed successfully with appropriate local wound care and secondary procedures, without complete flap loss (Figure 4).

Case 5

A 40-year-old male patient presented with a history of an RTA and an exposed tibia with a soft tissue defect of size 12 x 5 cms at the lower third of the right leg. On colour Doppler, there was monophasic flow in the ATA and PTA. Inferiorly based cross-leg flap was harvested based on PTA perforators in the normal leg. An external fixator was applied with the right knee slightly flexed, and the bilateral lower limbs were supported with a frame.

Case 6

A 36-year-old male patient presented with a segmental loss of tibia at the mid and lower third of the left leg with a soft tissue defect of size 14 x 7 cms following an RTA. On colour Doppler, there was monophasic flow in the ATA and the DPA. A transverse cross-leg flap was done based on PTA perforators, and an external fixator, along with the frame, was applied for immobilization for three weeks. Subsequently, division and insetting of the flap were done. We noticed distal flap necrosis of 2 cms, which was managed successfully with appropriate local wound care and secondary procedures, without complete flap loss.

Case 7

A 46-year-old male patient sustained an RTA with exposed left calcaneum, closed fracture of the tibia and fibula. On colour Doppler, there was monophasic flow in the ATA and PTA. An inferiorly based cross-leg flap was done based on the PTA perforators from the opposite leg for a soft tissue defect of size 10 x 6 cms. Postoperative stabilisation was done with an external fixator with a frame by flexing the left knee and keeping the left heel near the right lower leg.

Case 8

A 28-year-old male patient presented with post-traumatic exposed fractured tibia at the junction of the mid to lower third of the right leg with a soft tissue defect of size 15 x 6 cms with monophasic flow in the ATA and DPA. A transverse cross-leg flap was done, and an external fixator was applied for immobilization along with the frame. The delay started after three weeks, and division-insetting of the flap was finally done.

## Discussion

In the cases assessed in this study, additional elevation rods were incorporated into the conventional bilateral biplanar external fixator construct. This modification offers several practical advantages beyond limb immobilization. The elevated configuration suspends both lower limbs above the bed surface, thereby minimizing prolonged pressure over the posterior heels and reducing the risk of pressure-related skin injury. It also prevents direct contact of the limbs with the bed, reducing moisture accumulation and skin maceration. Furthermore, the elevated construct provides unobstructed access to both the flap and donor sites, allowing dressing changes, flap monitoring, and wound inspection to be performed without repeated manual limb elevation or manipulation. This may improve the ease of postoperative care, enhance patient comfort, and minimize unnecessary movement that could potentially compromise flap stability during the immobilization period. Although these practical advantages require confirmation in prospective comparative studies, the technique offers a simple and reproducible modification that can be readily incorporated into existing external fixation constructs.

Since the introduction of the free flap in 1970, microsurgical reconstruction has become the gold standard for lower extremity soft tissue defects, and indications for the cross-leg flap have consequently declined [6]; these flaps are considered backup options. Nevertheless, lower extremity trauma and infection remain significant health burdens in low- and middle-income countries, where delayed presentation and limited microsurgical infrastructure continue to restrict free flap access [7]. Microsurgical free flap is the best choice for extensive trauma of the lower extremities, but the success of free flap depends on the availability of a good recipient vascular status [8]. Hamilton first introduced the cross-leg flap in 1854 [9]. During the Second World War, this flap was used extensively with good results. Stark (1950) standardized the procedure and summarized its usefulness for trauma of the lower extremities [10].

Prior to Ponten's inclusion of the deep fascia [11], cross-leg flaps were raised as random-pattern flaps with a length-to-width ratio of 1:1. Following the incorporation of the deep fascia (fasciocutaneous design), flaps with a length-to-width ratio of up to 3:1 can reliably be raised. Various reconstruction methods have been developed to cover defects of the lower extremities. However, the use of free flaps and local pedicle flaps has limitations in patients with severe blood flow insufficiency after substantial artery injury. Two-vessel injury, non-availability of local tissue due to skin avulsion, and muscle loss along with vascular injury are indications for a cross-leg flap, such as trivial trauma that causes extensive soft tissue injury with segmental loss of bone, making the local flaps unavailable, and trauma with Gustilo-Anderson 3C injury, in which it is difficult to do a free flap [12].

The cross-leg flap resurfaces as a plausible choice in these circumstances. Cross-leg flaps can be pedicled flaps from the opposite leg or free cross-leg flaps by anastomosing the donor vessel to one of the vessels of the uninjured leg. Free cross-leg flaps were done to decrease morbidity from the normal leg. A free cross-leg Latissimus Dorsi muscle flap was done by end-to-side anastomosis in a mutilated leg by Abdelmegeed et al. [13]. Zhou et al. did a modified donor blood flow-preserving cross-leg anterolateral thigh flap for complex lower extremity reconstruction. They used the PTA as a donor vessel in all 22 of their patients with complex trauma below knee level [14]. For pedicled cross-leg flaps, PTA perforator-based flaps were more commonly done by Galwa et al. [15]. Conventionally, surgeons prefer plaster of Paris (POP) slab for stabilisation and immobilisation of two limbs, but external fixation is a good option to prevent flap dehiscence [16]. Although POP immobilization remains a simple and cost-effective method for cross-leg flap stabilization, external fixation offers the advantages of rigid immobilization, unrestricted access to both the flap and donor sites for dressing and monitoring, and easier maintenance of limb position. The present modification further enhances these advantages by elevating the limbs, thereby facilitating postoperative wound care and reducing pressure-related complications. Additional vertical tubular rods help in elevating the limbs off the bed and prevent ulcers, maceration, and ease of dressing [17].

The addition of elevation rods to the external fixator construct maintains both lower limbs in a suspended position, preventing direct heel contact with the bed surface. By off-loading the posterior heels, the construct reduces sustained pressure over bony prominences and may decrease the risk of pressure-related skin injury. This mechanical off-loading, together with routine nursing measures such as regular skin inspection and appropriate padding, contributes to the prevention of heel pressure sores during the prolonged immobilization required for cross-leg flap reconstruction. There will be an increased tendency to develop pressure ulcers at vulnerable points like the calcaneum, pelvis, and abnormal sites like the dorsum of the foot and ankle [5]. Cross-leg flap needs thoughtful preoperative planning for the type of flap, position of limbs, and type of fixation needed.

In all our eight cases, perforators were marked preoperatively, and thorough planning was done for the position of the limbs. In all eight cases, the defect involved the mid-third to lower third of the leg and hindfoot, with varying degrees of bony exposure, and composite defects were also included. The cross-leg flap was selected in seven patients because significant vascular compromise of the injured limb rendered local or free flap reconstruction unsuitable or high risk. In one patient, the procedure was undertaken as a salvage option following necrosis of a reverse sural artery flap, which exhausted the available local reconstructive options. Double elevation is needed for flaps, including the mid to lower third of the leg; single elevation of the external fixator is enough for the flap at the foot. Placing a wooden plank under the vertical external fixator helps in preventing the cushioning effect on the bed, which causes the fixator to dip down and makes the limb come into contact with the bed. In two patients with partial flap loss, these complications were most likely attributable to patient- and wound-related factors, including compromised vascularity and the severity of the initial soft tissue injury, rather than failure of the modified external fixator construct. Both cases were managed successfully with appropriate local wound care and secondary procedures, without complete flap loss.

Although external fixation provides rigid immobilization and facilitates flap monitoring and wound care, it is associated with certain disadvantages compared with POP immobilization. These include the requirement for an operative procedure for pin placement, the risk of pin tract infection, higher cost, dependence on specialized equipment and surgical expertise, and potential patient discomfort related to the external frame. Therefore, the choice between external fixation and POP immobilization should be individualized based on patient characteristics, flap requirements, and surgeon preference. The present study focuses on optimizing postoperative management in cases where external fixation is already considered the preferred method of immobilization.

Prospective comparative studies evaluating the modified external fixator against conventional POP immobilization or standard external fixation, using standardized objective outcome measures, are warranted to establish its comparative effectiveness.

Limitations of this study

This is a retrospective study involving a small number of patients (n=8) without a control group and with the heterogeneity of patient characteristics, including defect size, aetiology, vascular status, flap type, and timing of reconstruction, which precludes direct comparison with conventional immobilization techniques such as standard external fixation or POP immobilization. The standardized objective outcome measures, including dressing change time, pain scores, quantitative assessment of maceration, pressure-related complications, time to flap division, pin-tract infection rates, validated functional outcome measures, and patient-reported comfort, were not prospectively recorded. The retrospective single-center design introduces the possibility of selection bias. Therefore, the findings should be interpreted as demonstrating the technical feasibility and clinical applicability of the proposed modification rather than establishing its superiority. Prospective comparative studies with larger cohorts and standardized outcome assessment are warranted to validate these preliminary observations.

## Conclusions

External fixators play a crucial role in the success of the cross-leg flap procedure by providing stability, immobilization, and optimal positioning. By understanding the benefits and types of external fixators, surgeons can make informed decisions about their use in reconstructive surgery, ultimately improving patient outcomes and promoting optimal healing. Incorporation of this modification will surely help prevent pressure injuries over the heel and facilitate regular dressing changes, and avoid maceration and pressure ulcers. While objective outcome data remain limited in this series, the modification is simple, low-cost, and reproducible, and warrants prospective validation in larger cohorts.
